# The Influence of Science Technology Engineering Arts Mathematics-Based Psychological Capital Combined With Ideological and Political Education on the Entrepreneurial Performance and Sports Morality of College Teachers and Students

**DOI:** 10.3389/fpsyg.2022.911915

**Published:** 2022-06-28

**Authors:** Ying Jin

**Affiliations:** School of Physical Education and Health, Wenzhou University, Wenzhou, China

**Keywords:** psychological capital, Ideological and Political Education (IPE), entrepreneurial performance, sports ethics, employment

## Abstract

This study aims to alleviate the current tense employment situation and study the entrepreneurial situation of teachers and students in colleges and universities. Firstly, based on the educational concept of Science Technology Engineering Arts Mathematics (STEAM), Ideological and Political Education (IPE) is added to psychological capital to explore the effect of the combination of the two on entrepreneurial performance. An entrepreneurial performance impact model is constructed, and the questionnaire is set. Secondly, the influence of psychological capital combined with IPE on sports morality is explored, and a sports morality questionnaire is designed. Finally, the questionnaire results are analyzed. The results showed that most of the participants in the survey are under the age of 25. The 25- to 29-year-olds are the smallest among those surveyed. The respondents who participated in the survey had the most undergraduate degrees, accounting for 43.4%. The 40 items on entrepreneurial performance this time obey a normal distribution, and the scale has good reliability and validity. The main factor analysis results obtained by principal component analysis include 6 factors. Their total explanatory power exceeds 67%, and the six factors screened out this time are well represented. The model tolerance is between 0.45 and 0.5, and the Variance Inflation Factor (VIF) value is less than 10. The scale does not suffer from multi-collinearity issues. IPE significantly strengthens the effect of psychological capital on entrepreneurial performance. The influences of various sports on sportsmanship, bravery, self-confidence, and self-transcendence are significantly different, and the *P* value is less than 0.001. The difference in aggressiveness is less than 0.01, indicating that it is very significant. Different sports have significant differences in the cultivation of sports morality, and the *P* value is less than 0.05. The differences in self-discipline are also significant, and there are extremely significant differences in compliance with rules, and the *P* value here is less than 0.001. There are no significant differences in sporting qualities. IPE combined with psychological capital has a significant impact on entrepreneurial performance. These contents provide references for the entrepreneurship education of teachers and students in colleges and universities. The contribution lies in expanding the research on psychological capital in entrepreneurial performance and laying a foundation for the combination of psychological capital and IPE.

## Introduction

Science Technology Engineering Arts Mathematics (STEAM) education originated in the United States. The purpose of STEAM education is to develop engaged and globally competitive citizens. In recent years, STEAM education has been seen as a powerful way to cultivate innovative talents (Ephrem et al., 2021). In 2016, China added STEAM education to an important means of cultivating innovative talents. STEAM education involves multiple disciplines. It aims to integrate content from various fields such as art, engineering, technology, science, and mathematics, and emphasizes the organic combination of humanistic literacy and scientific spirit (Adel, 2021). There are few studies on the application of STEAM education in Chinese higher education. Under the current situation of innovation and entrepreneurship, the application of STEAM to entrepreneurship research in higher education will help improve the current situation of entrepreneurship education for college students. The application of STEAM education concept is reflected in the combination of psychological capital, Ideological and Political Education (IPE), and sports ethics.

With the expansion of Chinese universities, there are more and more college graduates, and the employment environment for Chinese college students is becoming increasingly severe. One of the ways to alleviate this situation is college students’ entrepreneurship. Compared with developed countries, the proportion of college students who choose to start a business in China is relatively small (Danioni et al., 2021). Therefore, it is very necessary for colleges and universities to carry out entrepreneurship education. The quality of entrepreneurship education is generally measured by entrepreneurial performance (Grözinger et al., 2022). Since the environment of the research object is higher education, college teachers are included in the research object. Based on STEAM education, this study introduces psychological capital to study the entrepreneurial performance of college students. Psychological capital refers to a positive psychological state exhibited by individuals in the process of growth and development (Gao et al., 2021). It is the core psychological element that exceeds social capital and personal capital, and is a psychological resource that promotes personal growth and performance improvement (Feng et al., 2021). The research on psychological capital applied to college students’ entrepreneurship has gradually increased in recent years. There are many intervention studies and status quo investigations, but there is a lack of empirical research.

Based on alleviating the current employment pressure and vigorously promoting innovation and entrepreneurship, this study is based on the STEAM concept and introduces college students’ IPE and physical education. The sports morality of college students’ values, world outlook and outlook on life is the way of expression, and the entrepreneurial performance of teachers and students in colleges and universities is studied. Psychological capital and IPE are used to study sports morality. The core of the research is the influence of the dual effects of psychological capital and IPE on the entrepreneurial performance of college teachers and students under STEAM education. The innovation is to introduce IPE and psychological capital into the study of entrepreneurial performance and analyze the influence of their joint action on sports morality. Firstly, the related theories and research status of psychological capital and entrepreneurial performance are analyzed. Secondly, a theoretical model and a hypothetical model of the impact of psychological capital combined with IPE on entrepreneurial performance are constructed, and a questionnaire on the impact of IPE and psychological capital on the sports morality of college teachers and students is designed. Finally, data processing is carried out by combining logic analysis and mathematical statistics. The research puts forward the direction and reference for the improvement of entrepreneurship education in colleges and universities.

## Literature Review

The research focuses on the influence of psychological capital on entrepreneurial performance. Therefore, the literature review part will focus on psychological capital and entrepreneurial performance.

Psychological capital is first measured by two dimensions, the point of control and self-esteem. As researchers do not explore, psychological capital measures develop toward a multidimensional structure (Hindman et al., 2021). At present, the widely used psychological capital questionnaire is proposed by the former president of the American Management Association based on positive psychology by Luthans. It contains four dimensions, namely, self-efficacy, optimism, hope, and resilience (Hatak et al., 2021). Based on the Chinese cultural background, Chinese scholars have carried out local development of psychological capital. At present, psychological capital research mostly focuses on individuals, and the research direction basically tends toward psychology (Lee, 2021). The starting point of psychological capital is to take enterprise employees as the object, the background is the work situation, and the purpose is to obtain the influence of psychological capital on employee job satisfaction and job performance based on human resource management (Kostorz and Sas-Nowosielski, 2021). The research on psychological capital in China started relatively late, but the research background and purpose are basically the same as those in Western countries (Hunter-Doniger, 2021). At present, the research object of psychological capital has gradually shifted from enterprise employees to students, and the role of psychological capital in the employment of students has become a new research hotspot (Hurst, 2021). Psychological capital can predict student performance, academic achievement level, employability, etc. It can also predict student mental health, emotion regulation, and emotion management (Yaffe et al., 2021).

Due to differences in viewpoints, the current research on entrepreneurial performance can be divided into four aspects, namely, entrepreneurial external environment and opportunities, entrepreneurial resources, entrepreneurial strategy choices, and internal entrepreneurial environment (Liu et al., 2021). These four aspects are summarized under the four theoretical foundations of population ecology theory, resource-based theory, strategic adaptation theory, and social cognition theory. Population ecology theory believes that the influencing factors of entrepreneurial performance are environmental resource density and entrepreneurial organization. The external environment of entrepreneurship, as a repository of entrepreneurial resources, is like the law of biological evolution. Entrepreneurial organizations are analogized to a biological population whose ability to adapt to the external environment is the key to survival (López et al., 2021). The theory of strategic adaptation believes that the strategic choice of entrepreneurial organizations is greater than the external resources and internal resources of entrepreneurial enterprises. The formulation of entrepreneurial strategies directly affects the results of value creation by entrepreneurial organizations (Upadhyay et al., 2021). Enterprises can develop different strategies and create different performance levels through their own changes (Qi et al., 2021). The resource conservation theory argues that entrepreneurial organizations need to regard the entrepreneurial process as the main factor affecting entrepreneurial performance. The entrepreneurial process is the process of the entrepreneurial organization integrating social resources. Effective use of advantageous resources is conducive to improving entrepreneurial performance (Mangenda Tshiaba et al., 2021). The social cognitive theory believes that the behavior and characteristics of entrepreneurial organizations lead to different entrepreneurial performances. Entrepreneurs should pay more attention to their own ability development and trait development (Xu et al., 2021).

Based on these studies, the research on psychological capital is mostly carried out at the theoretical level, and it is less integrated with other theories. The results of entrepreneurial performance show that the influencing factors of entrepreneurial performance are not single. Therefore, based on the STEAM concept, IPE is introduced to strengthen psychological capital, and select survival indicators and growth indicators as the measurement indicators of entrepreneurial performance. Finally, psychological capital is combined with the impact of IPE on the entrepreneurial performance of college teachers and students.

## The Influence of Psychological Capital Combined With Ideological and Political Education on Entrepreneurial Performance and Sports Morality

### The Effect of Psychological Capital Combined With Ideological and Political Education on Entrepreneurial Performance

This study aims to combine psychological capital better and introduce the theory of resource conservation. Resource conservation theory argues that individuals can alleviate emotional exhaustion due to stress by continually acquiring, maintaining, and protecting their valuable resources. One of the important resource classifications in resource conservation theory is an individual’s stress-resilience potential, that is, positive personality traits such as self-confidence, optimism, and self-efficacy (Zhang and Li, 2021). Resource conservation theory has been widely used in the influencing factors of entrepreneurial performance. Entrepreneurs need to integrate and utilize resources in the process of starting a business, including external social capital, stable personality traits, and characteristic resources for learning and development (Luo et al., 2021). The theory of psychological capital development believes that the development of individual psychological capital should be carried out by cultivating qualities such as self-efficacy, resilience, optimism, and hope (Rajah et al., 2021).

**FIGURE 1:**
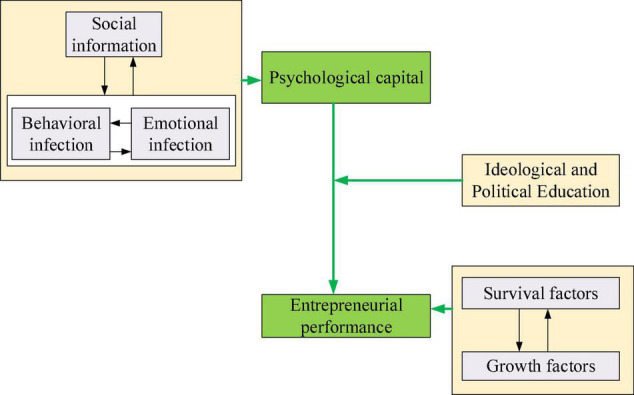
Theoretical model of the impact of psychological capital on entrepreneurial performance.

If entrepreneurs can integrate the positive psychological resources of their own strengths, preserve, and utilize them, they can bring their strengths into play in entrepreneurial performance (Welter and Scrimpshire, 2021). According to the basic theoretical analysis, one of the important factors affecting the level of entrepreneurial performance is the degree of resource preservation, and psychological capital is an important resource that can be preserved and utilized. Based on the STEAM concept and university IPE, a theoretical model of the impact of psychological capital on entrepreneurial performance is proposed, as shown in Figure 1.

In Figure 1, behavioral contagion and emotional contagion are affected by college IPE. Among them, IPE plays a strengthening role.

**FIGURE 2:**
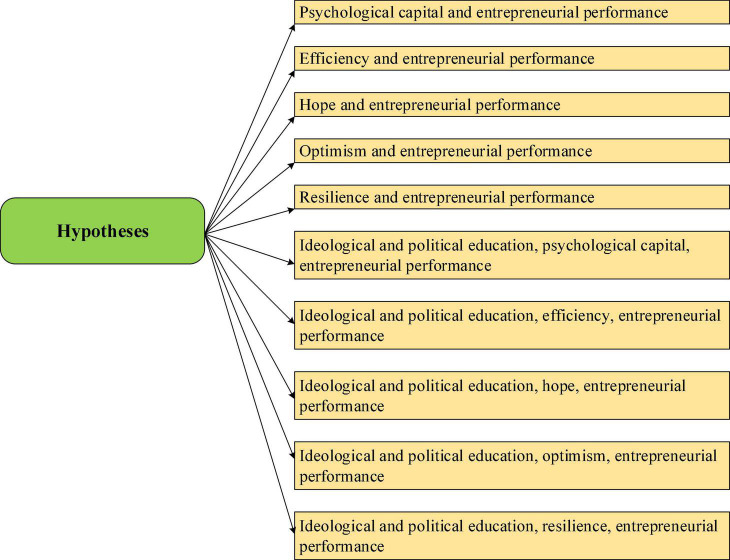
Hypotheses of factors influencing entrepreneurial performance.

Psychological capital is generated in the context of positive psychology, which makes up for the deficiency of human capital theory. The influence of psychological capital on entrepreneurial performance will be more obvious under the reinforcement of IPE. Confidence has a positive effect on the improvement of entrepreneurial performance. The positive effects of optimism, self-efficacy, and goals are verified in entrepreneurial performance improvement. At present, research focuses on the relationship between the components of psychological capital and entrepreneurial performance. Combined with previous research (Singh, 2021), the positive effects of four dimensions of psychological capital, efficacy, hope, optimism, and resilience are explored in improving entrepreneurial performance. These four dimensions are also key parts of IPE reinforcement. The overall hypothesis is proposed due to the strengthening effect of IPE, as shown in Figure 2.

In Figure 2, a total of 10 hypotheses are set, and the overall focus is on the impact of the strengthening function of psychological capital and IPE on entrepreneurial performance. All assumptions are positive.

**FIGURE 3:**
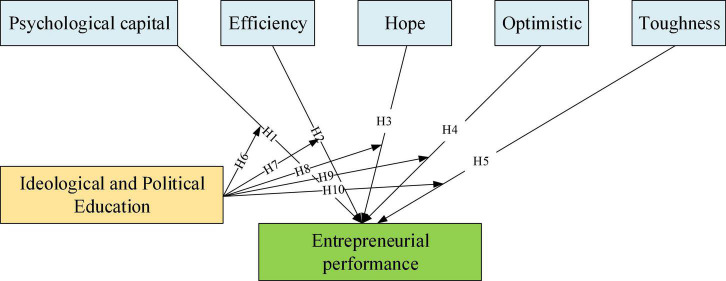
Model of the impact of IPE strengthening psychological capital on entrepreneurial performance.

A model of the impact of IPE-enhanced psychological capital on entrepreneurial performance is constructed based on the content, as shown in Figure 3 (Poolsawat, 2021).

In Figure 3, the relationship between the influencing factors of entrepreneurial performance is given, which is more convenient for the setting of the following items. English initials replace the items in Figure 3. “PC” is used for psychological capital, “E” is effectiveness, “H” is hope, “O” is optimism, “T” is resilience, “IPE” is IPE, and “EP” is entrepreneurial performance.

The questionnaire on the influencing factors of entrepreneurial performance is divided into four parts, the background information of the participants, the psychological capital scale, the entrepreneurial performance scale, and the IPE impact scale. The basic information includes the gender, age, etc. of the respondents. The psychological capital scale contains four dimensions, each of which includes six items. The scale is designed based on previous research (Yang, 2021). The IPE Impact Scale includes six items, namely, IPE’s reinforcement of optimism, IPE’s reinforcement of hope, IPE’s reinforcement of effectiveness, IPE’s reinforcement of resilience, IPE’s direct effect on entrepreneurial performance, and IPE’s positive effect (Su et al., 2021). The results are shown in Table 1.

**TABLE 1:** Psychological capital scale.

Dimension	Measurement item	Number
Efficacy	Able to actively solve problems	E1
	Understand self-ability	E2
	Understanding entrepreneurial strategies	E3
	Able to accomplish work goals	E4
	Able to connect with customers	E5
	Able to deal with the problems encountered in entrepreneurship perfectly	E6
Hope	When you encounter difficulties, you can find a way to get out of it	H1
	Energetic	H2
	Confidence	H3
	Entrepreneurship is progressing smoothly	H4
	Able to find ways to achieve work goals	H5
	In the process of achieving the goal	H6
Optimism	Have a good vision for the bad	O1
	Make it clear that mistakes are inevitable	O2
	Can see the light at work	O3
	Remain optimistic	O4
	Things are not going the way you expected	O5
	When you encounter difficulties, believe that you can solve them	O6
Toughness	Difficulty moving forward when faced with setbacks	T1
	Courage to solve problems	T2
	Solve problems independently	T3
	Not impatient under pressure	T4
	Can survive tough times	T5
	Can handle many things at the same time	T6

The measurement dimensions in the Entrepreneurial Performance Scale are growth indicators and survival indicators, and the scores are scored using the Likert scale (Zheng and Ke, 2020). The Entrepreneurial Performance Scale consists of two dimensions and ten specific items. The items of the survivability indicator include the company’s profitability, financial distress, operating status, and operating time. Growth indicators include the number of employees in the company, the pace of new product development, sales growth, net income growth, market share growth, and company size (Valekh, 2021).

A reliability and validity test validate the questionnaire. The data analysis of the questionnaire is carried out using Statistical Product and Service Solutions (SPSS) 25.0, including descriptive analysis, correlation analysis, reliability and validity analysis, and regression analysis. Firstly, a descriptive analysis is performed based on the first part of the questionnaire. Secondly, the data are screened by reliability and validity analysis combined with principal component analysis. Finally, correlation analysis and regression analysis are obtained. The calculations involved in reliability analysis and validity analysis are shown in Eqs 1–3 (Ye and Chen, 2021).


(1) $$\mathrm{KMO}=\frac{\text{S}}{M+S}$$

Equation 1 represents the Kaiser–Meyer–Olkin (KMO) test. Among them, S refers to the sum of squares of all correlation coefficients between variables, and M is the sum of squares of partial correlation coefficients.


(2) $${\text{x}}^{2}=-\left[n-\left(2\,p+11\right)/6\right]\,\ln\,\left|R\right|$$

(3) df=p⁢(p-1)/2

Equations 2, 3 are the Bartlett sphericity test equations. n is the number of data records. p is the number of variables for factor analysis. “ln| R|” is the natural logarithmic function. |R| is the value of the determinant of the correlation coefficient matrix R.

The Variance Inflation Factor (VIF) in regression analysis is calculated as Eq. 4 (Xia, 2021).


(4) $$\text{VIF}=\frac{1}{1-{\text{R}}^{2}}$$

The larger the variance inflation coefficient VIF, the greater the possibility of collinearity between independent variables.

Tolerance is the inverse of VIF. Therefore, the calculation of tolerance H is as shown in Eq. 5.


(5) $$\text{H}=1-{\text{R}}^{2}$$

Teachers and students in a university entrepreneurial base are taken as the research objects. Members of the group have set up several entrepreneurial teams in a wide range of fields, including electronic information, biology, new medicine, aerospace, new materials, high-tech services, new energy and energy conservation, resources and environment, and advanced manufacturing and automation. The entrepreneurship rate of teachers and students in entrepreneurial bases of colleges and universities is high. It is influenced by IPE and sports moral education in colleges and universities, which has important research value. Here, the questionnaires are distributed among teachers and students of a university entrepreneurial base, and a total of 200 questionnaires are distributed. Distribution methods include on-site distribution, telephone questionnaires and mail questionnaires. This time, 183 valid questionnaires are recovered, and the questionnaire efficiency is 91.5%. Missing questions are considered invalid. Compared with the questionnaire efficiency of 90.6% in the research done by Weng et al. (2022), the latest research on entrepreneurial performance, the questionnaire efficiency of this study is higher.

### The Influence of Psychological Capital Combined With Ideological and Political Education on Sports Morality

Sports morality is the ideological and moral quality of individuals in sports. Physical education for teachers and students in colleges and universities requires participants to devote themselves to it, and add certain ideological and political elements, so that participants can accept IPE during the activity and cultivate good morals and behaviors. Adding IPE elements to physical education can not only improve students’ psychological quality, but also cultivate students’ lofty moral quality and sound personality, which has a huge impact on the formation of participants’ world outlook, values, and outlook on life. Both psychological capital and IPE focus on “moral education.” Therefore, this has a certain impact on the cultivation of physical integrity of teachers and students in colleges and universities. Most of the existing research is in the theoretical stage, and there are few studies on the joint effect of IPE and psychological capital in sports morality.

The questionnaire is designed based on the existing literature and expert opinions. The research object of this part is still the teachers and students of the university entrepreneurial base in the previous section. This part of the questionnaire will be distributed together with the questionnaires in the previous section, and the distribution method is the same as that in the previous section for the research to proceed smoothly. Therefore, the number of participants in this part of the survey is still 200. Due to the integrity of the questionnaire, the effective rate will be discussed as a whole, and the effective rate of the questionnaire is still 91.5%.

In this part of the questionnaire design, the reference scales include the Rosenberg self-esteem scale, the adolescent self-transcendence scale, the self-confidence scale, and the willpower scale. The questionnaire design process should try to include the four basic dimensions of psychological capital and refer to the content of IPE. The questionnaire divides sports morality into three dimensions, namely, sportsmanship, sportsmanship, and sportsmanship. Among them, sportsmanship includes self-esteem, self-confidence, courage, tenacity, self-transcendence, and aggressiveness. Sportsmanship includes following the rules, integrity, self-discipline and fairness and justice. Sports character includes civility and courtesy, mutual respect, teamwork, social responsibility, and a correct view of winning and losing. There are a total of 40 questions, the first 39 questions correspond to 15 indicators, and the last question is the criterion of the validity of the questionnaire.

This part of the questionnaire validity test is carried out by the expert evaluation method. The validity of the questionnaire was scored with reference to relevant expert opinions. Questionnaire validity is scored. A five-point scoring method is adopted, and the scores from high to low are very effective, effective, average, not very effective, and ineffective. The content of scoring includes questionnaire structure design, content design and overall design. Questionnaire validity scoring results will be given in the Section “Results and Discussion”. The reliability test of this part of the questionnaire is carried out by the “retest method.” Thirty college teachers and students are selected when they are distributed for the first time. The second round of questionnaire survey is conducted on this part of the respondents 14 days later. The two results are entered into SPSS software for analysis, and the correlation coefficient is finally obtained. This part of the questionnaire data analysis using SPSS software. Data processing includes mean, standard deviation, independent sample *T* test, one-way ANOVA, etc. This part of the questionnaire is scored on a five-point scale.

## Results in the Analysis of the Influencing Factors of Entrepreneurial Performance and Sports Ethics

### Descriptive Statistics

**FIGURE 4:**
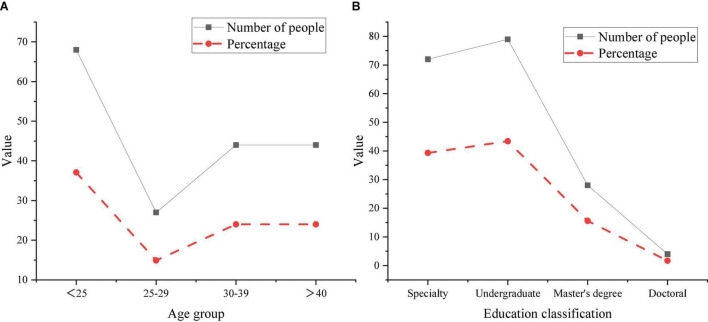
Descriptive statistical results [panel **(A)** is different ages and panel **(B)** is different educational backgrounds].

Among the investigators, 56.2% are male, and 43.8% are female. The descriptive statistics of education and age are shown in Figure 4.

In Figure 4, among the participants in this survey, the majority are under the age of 25, and the least are between the ages of 25 and 29. The number of respondents who participated had the largest number of undergraduates, accounting for 43.4%, followed by junior colleges, accounting for 39%. The number of master’s and doctoral students is relatively small. The research objects are college teachers and students. Therefore, the proportion of the population and the distribution of educational background are basically consistent with the overall level of the research subjects. Feng et al. (2022) found that in a study on the characteristics of entrepreneurs, most entrepreneurs were over 30 years old. The results are different from those here. This conclusion may be due to the small sample size of the study subjects from the same university and teachers (Feng et al., 2022).

### Result Analysis of Factors Influencing Entrepreneurial Performance

**FIGURE 5:**
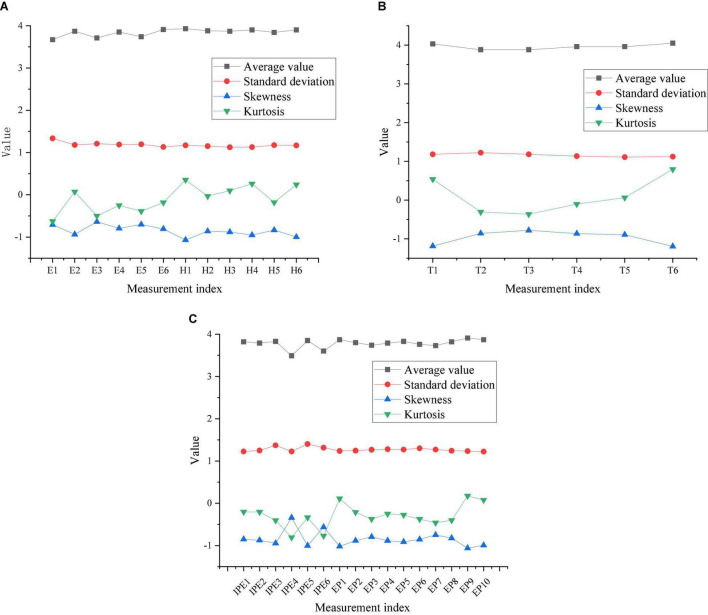
Results of measurement indicators of the Entrepreneurial Performance Impact Scale [panel **(A)** is efficacy and hope results, panel **(B)** is optimism and resilience results, and panel **(C)** is IPE and entrepreneurial performance results].

The mean, standard deviation, skewness, and kurtosis of the items of the Entrepreneurial Performance Impact Scale are shown in Figure 5.

In Figure 5, the mean of the formal sample results is between 3.5 and 4.1, the standard deviation is between 1.1 and 1.4, the skewness is between –1.2 and –0.3, and the kurtosis is between −0.8 and 0.8. The basic requirements for the sample to meet the normal distribution are: the absolute value of kurtosis is less than 10, and the absolute value of skewness is less than 3. The 40 items of this time obey the normal distribution.

**FIGURE 6:**
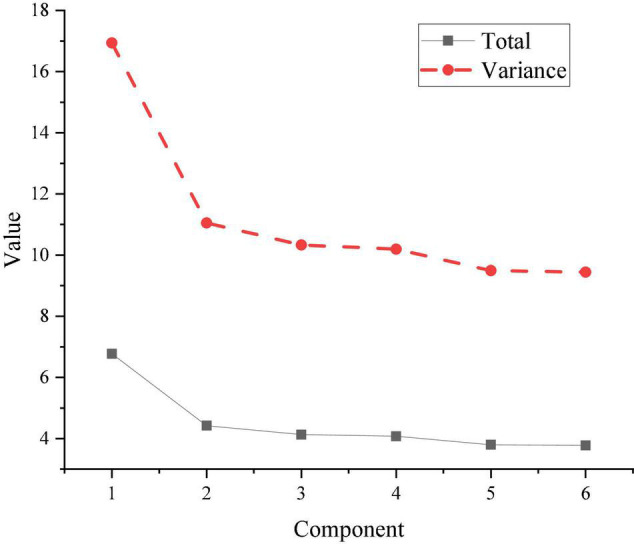
Extraction results of principal component analysis.

The Cronbach’s Alpha coefficients of the scale are analyzed by SPSS software, which are 0.89, 0.92, 0.88, 0.87, 0.91, and 0.95, all of which are greater than 0.7, indicating the reliability of each variable is good. The KMO value is 0.96, which is greater than 0.7, indicating that the scale has good validity. It is suitable for factor analysis. Six principal components are obtained based on principal component analysis. The result obtained after extracting the common factor rotation is shown in Figure 6.

In Figure 6, the factor analysis results include six factors, with explanatory power of 16.9, 11.1, 10.3, 10.2, 9.4, and 9.3%, respectively. Their total explanatory power exceeds 67% > 50%, indicating that the six factors screened out this time are well represented. The cross-loading of the component matrix obtained by rotation is less than 0.4, indicating that the scale has good construct validity.

**FIGURE 7:**
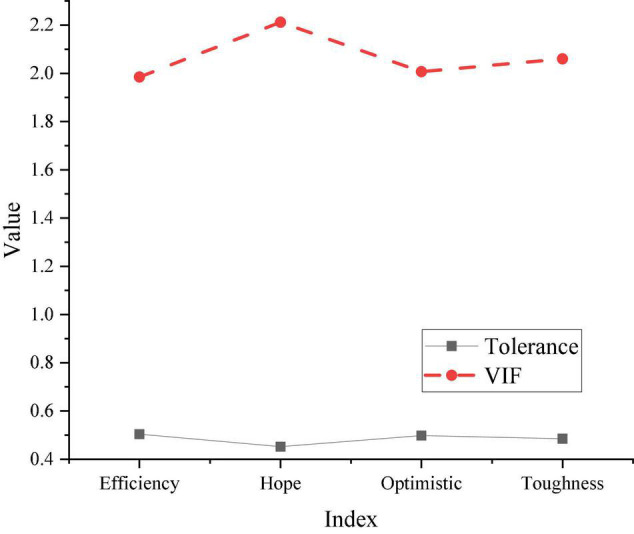
Regression analysis of psychological capital and entrepreneurial performance.

The correlation coefficients obtained by SPSS software are all between 0.4 and 0.65, which had a significant impact. The regression analysis results of psychological capital and entrepreneurial performance are shown in Figure 7.

In Figure 7, the model tolerance is between 0.45 and 0.5, and the VIF value is less than 10. The known tolerance value interval is [0, 1], so the scale does not have multi-collinearity problems. From the results of SPSS software, the significance in the model is less than 0.001, indicating that psychological capital has a significant regression effect on entrepreneurial performance. Hussain and Shahzad (2022) believed that psychological capital positively impacted entrepreneurial performance in their research on psychological capital and entrepreneurial performance.

**FIGURE 8:**
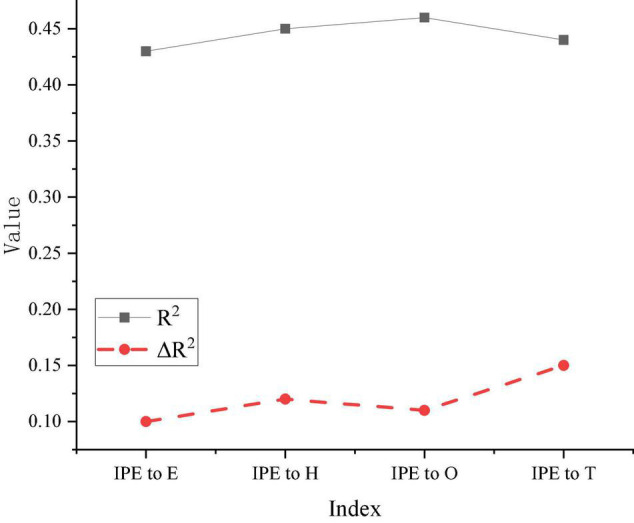
Results of reinforcement of IPE.

The IPE plays a strengthening role in the impact of psychological capital on entrepreneurial performance. Hierarchical regression is carried out by adjusting variables, and this time the results of different levels are treated. The result is shown in Figure 8.

In Figure 8, in the strengthening effect of IPE on the effect of efficacy on entrepreneurial performance, the regression coefficient is 0.43, and the regression coefficient is significant. Therefore, IPE has a reinforcing effect on the effect of efficacy on entrepreneurial performance. In the strengthening effect of IPE on the effect of hope in entrepreneurial performance, the regression coefficient is 0.43, and the regression coefficient is significant. The regression coefficients of IPE in optimism and resilience are still significant, indicating that IPE plays a significant role in enhancing the influence of psychological capital on entrepreneurial performance.

### Analysis of the Results of Influencing Factors of Sports Morality

**FIGURE 9:**
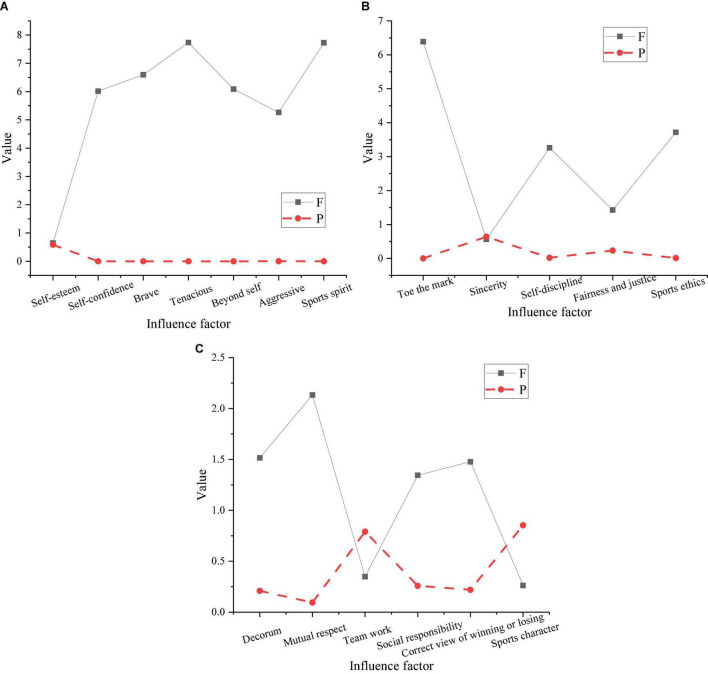
Sports morality affects scores [panel **(A)** is sports spirit, panel **(B)** is sports morality, and panel **(C)** is sports character].

Since there are many studies on sports morality in terms of the gender of teachers and students in colleges and universities, only the types of sports are analyzed here. The types of sports include skill-oriented same-field confrontation projects, skill-oriented net-separation competition projects, skill-oriented difficult-to-perform events, and physical fitness-oriented fast-strength events. Among them, various projects accounting for 36.1, 23.2, 29.8, and 10.9% of the total number of people, respectively. The impact scores and significant results of sports morality are shown in Figure 9.

In Figure 9, the influence of various sports types on sportsmanship, bravery, self-confidence, and self-transcendence is significantly different, the *P* value is less than 0.001, and the difference in aggressiveness is less than 0.01, indicating that it is very significant. Different sports have significant differences in the cultivation of sports morality, the *P* value is less than 0.05, the differences in self-discipline are also significant, and there are extremely significant differences in compliance with rules. The *P* value here is less than 0.001. There are no significant differences in sporting qualities.

## Discussion

The statistical results of the basic information of the respondents showed that most of them are younger than 25 years old. Since the respondents are teachers and students of entrepreneurial bases, they all have entrepreneurial experience by default in the questionnaire. Therefore, the people involved in entrepreneurship in the sample are younger, which is inconsistent with the latest research conclusion of Veselinovic et al. (2022) on entrepreneurs. Younger entrepreneurs may be more adventurous (Šebestová and Popescu, 2022). Older entrepreneurs tend to be more cautious when starting a business because of their family ties. Efficacy, optimism, hope, and resilience in psychological capital positively affect entrepreneurial performance. The research on the strengthening effect of IPE introduced shows that the regression coefficient of IPE on efficacy, hope, optimism, and resilience in psychological capital is less than 0.01, and the effect is significant. IPE has a strengthening effect on applying psychological capital in entrepreneurial performance. Chen (2022) showed that IPE positively affects individual psychological wellbeing. This conclusion is consistent with the results of this study. The effect of the entrepreneurial performance is added. In the study of sports morality, different types of sports have a greater impact on the aggressiveness in sports morality. Here, sportsmanship is divided into sportsmanship, morality, and character. This classification is consistent with Bou-Franch (2022).

## Conclusion

Based on the background of the low entrepreneurial rate of teachers and students in Chinese universities, psychological capital and IPE are introduced to study the improvement of entrepreneurial performance. The innovation lies in the introduction of IPE and psychological capital into entrepreneurial performance and the analysis of the influence of the two together on sports ethics. This study is divided into two parts, namely, the effect of psychological capital combined with IPE on entrepreneurial performance and the effect of psychological capital combined with IPE on sports ethics. The results show that psychological capital combined with IPE significantly impacts entrepreneurial performance. IPE plays a role in strengthening psychological capital; IPE combined with psychological capital significantly impacts sports morality, but not on sports morality. This study provides theoretical support for improving the entrepreneurial rate of teachers and students in colleges and universities. Due to the limited energy of the author, some deficiencies still exist. The research did not consider the impact of entrepreneurial experience on ability and performance; the teaching conditions were not studied; the data processing part of the entrepreneurial performance research was not detailed enough; in the morality of the sports research, only sports types were selected, which made the results relatively single. In the future, the influence of entrepreneurial experience and teaching conditions will be further increased. The data will be further analyzed and discussed. Factors such as age and entrepreneurial time will be added to increase the link between sports ethics and entrepreneurial performance. This study lays the foundation for the in-depth study of psychological capital in entrepreneurial performance.

## Data Availability Statement

The raw data supporting the conclusions of this article will be made available by the authors, without undue reservation.

## Ethics Statement

The studies involving human participants were reviewed and approved by Wenzhou University Ethics Committee. The patients/participants provided their written informed consent to participate in this study. Written informed consent was obtained from the individual(s) for the publication of any potentially identifiable images or data included in this article.

## Author Contributions

The author confirms being the sole contributor of this work and has approved it for publication.

## Conflict of Interest

The author declares that the research was conducted in the absence of any commercial or financial relationships that could be construed as a potential conflict of interest.

## Publisher’s Note

All claims expressed in this article are solely those of the authors and do not necessarily represent those of their affiliated organizations, or those of the publisher, the editors and the reviewers. Any product that may be evaluated in this article, or claim that may be made by its manufacturer, is not guaranteed or endorsed by the publisher.
