# Supplementary material for: The Influence of Science Technology Engineering Arts Mathematics-Based Psychological Capital Combined With Ideological and Political Education on the Entrepreneurial Performance and Sports Morality of College Teachers and Students
Source: Front Psychol. 2022 Jun 28;13:911915. doi: 10.3389/fpsyg.2022.911915 (PMC9275666; doi:10.3389/fpsyg.2022.911915)
Supplement: Supplementary file 1 [file Data_Sheet_1.docx]

**Appendix:**

Influence of STEAM-Based Psychological Capital Combined with Ideological and Political Education on Entrepreneurial Performance and Sports Morality of College Teachers and Students

Questionnaire

Dear Madam/Sir:

Thank you very much for your support and participation!

This survey is a special survey to study the impact of ideological and political education and psychological capital on entrepreneurial performance. The subjects of this survey are technical, entrepreneurial team members. We solemnly promise that the questionnaire results are only used for academic research. The whole process of the survey will be anonymous, and all the information you provide will be kept confidential and will not have any impact on your personal, team, and business.

**Part 1: Background Information (Choose the options you see fit, there is at most one option per question)**

1. Your gender is:

Female ( ) Male ( )

2. Your age is:

<25 years old ( ) 25~29 years old ( ) 30~39 years old ( ) >40 years old ( )

3. Your education is:

Junior college ( ) Undergraduate ( ) Master ( ) Doctoral ( )

**Part II: The Questionnaire of Psychological Capital**

Note: in this section, 1 means "totally disagree", 2 means "disagree", 3 means "neutral", 4 means "agree", and 5 means "totally agree". Please fill in according to the real situation. There is no right or wrong answer.

| item | totally disagree | disagree | neutral | agree | totally agree |
| --- | --- | --- | --- | --- | --- |
| E1: able to actively solve problems | 1 | 2 | 3 | 4 | 5 |
| E2: understanding self-abilities | 1 | 2 | 3 | 4 | 5 |
| E3: understanding entrepreneurial strategy | 1 | 2 | 3 | 4 | 5 |
| E4: able to complete work objectives | 1 | 2 | 3 | 4 | 5 |
| E5: ability to connect with customers | 1 | 2 | 3 | 4 | 5 |
| E6: can perfectly handle the problems encountered in entrepreneurship | 1 | 2 | 3 | 4 | 5 |
| H1: when you encounter difficulties, you can find ways to get out of it | 1 | 2 | 3 | 4 | 5 |
| H2: vibrant | 1 | 2 | 3 | 4 | 5 |
| H3: always be confident | 1 | 2 | 3 | 4 | 5 |
| H4: the current entrepreneurial work is progressing smoothly | 1 | 2 | 3 | 4 | 5 |
| H5: can think of ways to achieve work goals | 1 | 2 | 3 | 4 | 5 |
| H6: in the process of achieving the goal | 1 | 2 | 3 | 4 | 5 |
| O1: have a good vision for the bad | 1 | 2 | 3 | 4 | 5 |
| O2: make it clear that mistakes are inevitable | 1 | 2 | 3 | 4 | 5 |
| O3: can see the light at work | 1 | 2 | 3 | 4 | 5 |
| O4: remains optimistic | 1 | 2 | 3 | 4 | 5 |
| O5: things didn't go the way they were supposed to | 1 | 2 | 3 | 4 | 5 |
| O6: I believe it can be solved when encountering difficulties | 1 | 2 | 3 | 4 | 5 |
| T1: It is difficult to move forward when faced with setbacks | 1 | 2 | 3 | 4 | 5 |
| T2: courage to solve difficult problems | 1 | 2 | 3 | 4 | 5 |
| T3: Solve problems independently | 1 | 2 | 3 | 4 | 5 |
| T4: don't be impatient in the face of pressure | 1 | 2 | 3 | 4 | 5 |
| T5: can survive the tough times of starting a business | 1 | 2 | 3 | 4 | 5 |
| T6: can handle a lot of things at the same time | 1 | 2 | 3 | 4 | 5 |

**Part III: the Questionnaire of Entrepreneurial Performance**

Note: in this section, 1 means "totally disagree", 2 means "disagree", 3 means "neutral", 4 means "agree", and 5 means "totally agree". Please fill in according to the real situation. There is no right or wrong answer.

| item | totally disagree | disagree | neutral | agree | totally agree |
| --- | --- | --- | --- | --- | --- |
| EP1: the company has self-sustaining profitability | 1 | 2 | 3 | 4 | 5 |
| EP2: the company's operations did not encounter financial difficulties | 1 | 2 | 3 | 4 | 5 |
| EP3: the company is operating well | 1 | 2 | 3 | 4 | 5 |
| EP4: the company will continue to operate for more than ten years | 1 | 2 | 3 | 4 | 5 |
| EP5: the number of employees in the company is growing rapidly compared to its competitors | 1 | 2 | 3 | 4 | 5 |
| EP6: the company's new product or service is developing rapidly compared to its competitors | 1 | 2 | 3 | 4 | 5 |
| EP7: the company's sales are growing faster than its competitors | 1 | 2 | 3 | 4 | 5 |
| EP8: the company's net income is growing faster than its competitors | 1 | 2 | 3 | 4 | 5 |
| EP9: the company's market share is growing faster than its competitors | 1 | 2 | 3 | 4 | 5 |
| EP10: the company is expected to continue to grow | 1 | 2 | 3 | 4 | 5 |

**Part IV: Questionnaire on Ideological and Political Education**

Note: in this section, 1 means "totally disagree", 2 means "disagree", 3 means "neutral", 4 means "agree", and 5 means "totally agree". Please fill in according to the real situation. There is no right or wrong answer.

| item | totally disagree | disagree | neutral | agree | totally agree |
| --- | --- | --- | --- | --- | --- |
| IPE1: ideological and political education has a positive impact on your optimism | 1 | 2 | 3 | 4 | 5 |
| IPE2: ideological and political education has a positive impact on your mood of hope | 1 | 2 | 3 | 4 | 5 |
| IPE3: ideological and political education has a positive impact on your educational effectiveness | 1 | 2 | 3 | 4 | 5 |
| IPE4: ideological and political education has a positive impact on your resilience | 1 | 2 | 3 | 4 | 5 |
| IPE5: ideological and political education directly and positively affects your entrepreneurial performance | 1 | 2 | 3 | 4 | 5 |
| IPE6: ideological and political education has a positive impact on you | 1 | 2 | 3 | 4 | 5 |

Thank you for your participation.
